# Impact of Cardiometabolic Risk Factors and Steatotic Liver Disease on Liver‐Related Outcomes in Patients With Chronic Hepatitis C After Curative Antiviral Therapy

**DOI:** 10.1002/kjm2.70214

**Published:** 2026-04-22

**Authors:** Chung‐Feng Huang, Yi‐Hung Lin, Pei‐Chien Tsai, Ming‐Lun Yeh, Chih‐Wen Wang, Tyng‐Yuan Jang, Po‐Cheng Liang, Yu‐Ju Wei, Nai‐Jen Hou, Ming‐Yen Hsieh, Chao‐Kuan Huang, Tzu‐Chun Lin, Jee‐Fu Huang, Chia‐Yen Dai, Wan‐Long Chuang, Ming‐Lung Yu

**Affiliations:** ^1^ Hepatobiliary Division, Department of Internal Medicine and Hepatitis Center Kaohsiung Medical University Hospital, Kaohsiung Medical University Kaohsiung Taiwan; ^2^ School of Medicine and Hepatitis Research Center, College of Medicine, and Center of Metabolic Disorders and Obesity Kaohsiung Medical University Kaohsiung Taiwan; ^3^ Ph.D. Program in Translational Medicine, College of Medicine Kaohsiung Medical University Kaohsiung, and Academia Sinica Taiwan; ^4^ Department of Internal Medicine, Kaohsiung Municipal Siaogang Hospital Kaohsiung Medical University Kaohsiung Taiwan; ^5^ School of Medicine, College of Medicine and Center of Excellence for Metabolic Associated Fatty Liver Disease National Sun Yat‐sen University Kaohsiung Taiwan; ^6^ Department of Internal Medicine, Kaohsiung Municipal Gangshan Hospital Kaohsiung Medical University Kaohsiung Taiwan

**Keywords:** CHC, CMRF, HCC, SLD, SVR

## Abstract

Patients with chronic hepatitis C (CHC) frequently present with steatotic liver disease (SLD) and cardiometabolic risk factors (CMRFs). This study aimed to evaluate the impact of SLD and CMRFs on liver‐related outcomes (LROs) in CHC patients after HCV eradication. This study evaluated 21,972 CHC patients who received curative antivirals in Taiwan. LROs included newly developed hepatocellular carcinoma and liver decompensation. During a follow‐up period of 71,000 person‐years (PYs), 745 (3.4%) patients developed LROs (annual incidence of 1.05%). The annual incidence of LRO (136.2 vs. 80.7 per 10,000 PYs, *p* < 0.001) was significantly higher in patients without SLD than in those with SLD. Cox regression analysis revealed that SLD was independently associated with a lower risk of LRO (adjusted hazard ratio [aHR]/95% confidence intervals [CI]: 0.85/0.73–0.99, *p* = 0.038). There was an increased trend toward increased LRO risk in patients with a higher number of CMRFs than in those without CMRFs (aHR/CI: 1.35/1.02–1.78, 1.48/1.11–1.97, and 1.53/1.15–2.05 for 1, 2, and > 3 CMRFs, respectively). Non‐SLD patients who carried CMRFs were independently associated with a high risk of LROs compared to SLD patients without any CMRF carriage (aHR/CI: 1.97/1.11–3.50, *p* = 0.02). We concluded that CMRF burden had a dose‐dependent effect on LRO risk in CHC patients after curative antivirals. Non‐SLD CHC patients who possessed CMRFs were at a greater risk of LROs.

**Abbreviations**
ALT: alanine aminotransferase
AST: aspartate aminotransferase
CHC: chronic hepatitis C
CIs: confidence intervals
CMRFs: cardiometabolic risk factors
DAA: directly acting antiviral
DM: diabetes mellitus
eGFR: estimated glomerular filtration rate
FIB‐4: fibrosis index based on the 4 factors
HCC: hepatocellular carcinoma
HCV: hepatitis C virus
HRs: hazard ratios
HSI: hepatic steatosis index
KM: Kaplan–Meier
LC: liver cirrhosis
LRO: liver‐related outcome
MASLD: metabolic dysfunction‐associated steatotic liver disease
PYs: person‐years
SLD: steatotic liver disease
SVR: sustained virologic response

## 1 Introduction

Hepatitis C virus (HCV) infection has been a major public health threat to humans for decades and may lead to end‐stage liver disease and hepatocellular carcinoma (HCC). It is estimated that 31% of HCC cases are attributed to HCV infection worldwide [1]. HCV infection is also the major etiology of HCC, second to hepatitis B virus (HBV) infection, in Taiwan. Fortunately, HCV eradication using either interferon or directly acting antivirals (DAAs) based therapy greatly reduces the risk of liver‐related outcomes (LROs) and improves survival in Taiwanese patients [2, 3]. Nevertheless, owing to complex viral, host genetic, epigenetic and environmental interactions before or after HCV cure, HCC still occurs in a subset of patients with chronic hepatitis C (CHC) who have a sustained virological response (SVR) [4, 5]. The discrepant guidance of HCC surveillance post‐HCV eradication remains an unmet need in clinical care [6, 7].

Patients with HCV infection are prone to hepatic steatosis. The prevalence of hepatic steatosis in patients with HCV infection has been reported to be 35%–70%, which is higher than that reported in patients with other etiologies of liver diseases and in the general population [8, 9, 10, 11, 12]. The greater proportion of hepatic steatosis in patients with CHC may be attributed to insulin resistance, so‐called metabolic steatosis, or direct insult of virogenic steatosis of HCV genotype 3 [13]. Metabolic dysfunction is another extrahepatic manifestation of CHC. As hepatic steatosis has been proposed to be associated with liver disease progression or HCC development [14, 15, 16], metabolic disarrangement has been reported to overwhelm hepatic steatosis as the major risk factor for HCC in the post‐HCV curative status [17].

Steatotic liver disease (SLD) has been recently endorsed by international societies as an overarching term [18, 19]. Under this umbrella terminology, SLD further encompasses individuals with cardiometabolic risk factors (CMRFs), metabolic dysfunction‐associated steatotic liver disease (MASLD), and those without. We recently reported that CHC patients with metabolic steatosis who carry CMRFs may also be viewed as having HCV‐MASLD [20]. It has been proposed that CHC patients with MASLD are at greater risk of HCC than those without MASLD after HCV eradication [21]. As SLD may be negatively associated with liver disease severity late in the clinical course [22], whether the CMRF burden drives LROs in the presence of hepatic steatosis in CHC remains elusive. In this study, we aimed to address this issue by including CHC patients from two nationwide cohorts in Taiwan. We sought to evaluate the interactive impact of CMRF burden and the presence of SLD on LROs, including HCC and liver decompensation, in patients receiving curative antivirals.

## 2 Methods

### 2.1 Study Population

CHC patients were retrieved from two nationwide HCV registry cohorts in Taiwan: the Taiwanese Chronic Hepatitis C Cohort (T‐COACH) with interferon (IFN)‐based treatment and the TASL HCV registry (TACR) with DAA‐based treatment. These cohorts provided a major dataset of Taiwanese CHC patients receiving antivirals, representing approximately one‐fourth of the patients treated over the past two decades [23, 24, 25]. Patients were excluded if they had any of the following conditions: coinfection with hepatitis B virus or human immunodeficiency virus; a history of heavy alcohol consumption (> 20 g/day for women and > 30 g/day for men); unavailable data regarding SLD or CMRFs; failure to achieve a SVR (defined as undetectable HCV RNA throughout 24 weeks after the end of IFN‐based therapy or 12 weeks after the end of DAA therapy); liver decompensation, hepatocellular carcinoma or liver transplantation before treatment or occurrence of the outcomes of interest before achieving an SVR. The study was approved by the Institutional Review Boards of Kaohsiung Medical University Hospital, which adhered to the ethical standards of the Helsinki Declaration of 1975, revised in 2008. All patients provided written informed consent before study enrollment.

### 2.2 Measurements and Definitions of Variables

Biochemical analyses were conducted using a multichannel autoanalyzer (Hitachi Inc., Tokyo, Japan). The related clinical indices were calculated via standard methods: body mass index (BMI) = weight (kg)/height (m)^2^; fibrosis index based on four factors (FIB‐4) score = [age (year) × aspartate aminotransferase; AST (U/L)]/[platelet (×1000/μL) × alanine aminotransferase; ALT (U/L)^0.5^]. Estimated glomerular filtration rate (eGFR)= $186\times \text{creatinine}{\left(\mathrm{mg}/\mathrm{dL}\right)}^{-1.154}\times \;\mathrm{age}{\left(\text{year}\right)}^{-0.203}\times 0.742\;\left(\text{if female}\right)$. SVR was defined as undetectable HCV RNA at 24 weeks after IFN‐based therapies or 12 weeks after DAA‐based therapies. SLD was defined via ultrasonography by experienced hepatologists or a hepatic steatosis index (HSI) > 36 if untrasonography data was unavailable [26]. CMRFs included the following: (1) BMI ≥ 23 kg/m^2^; (2) fasting plasma glucose ≥ 100 mg/dL, glycated hemoglobin (HbA1C) ≥ 5.7% or a history of type 2 diabetes mellitus (DM) receiving treatment; (3) antihypertensive drug treatment; (4) triglycerides (TG) ≥ 150 mg/dL or lipid‐lowering treatment; and (5) high‐density lipoprotein cholesterol (HDL‐C) ≤ 40 mg/dL for men and ≤ 50 mg/dL for women or lipid‐lowering treatment [18, 19]. Patients with SLD without any CMRFs were defined as having simple SLD, whereas those with SLD who carried at least one CMRF were defined as having MASLD [20]. Liver cirrhosis was defined by any of the following: transient elastography (FibroScan; Echosens, Paris, France) > 12 kPa [27], acoustic radiation force impulse (> 1.98 m/s) [28] or the presence of clinical, radiological, endoscopic, or laboratory evidence of cirrhosis and/or portal hypertension. Definitions for hypertension, cerebrovascular disease, and cardiovascular disease relied on pre‐designed registry questionnaires. The definitions for chronic kidney disease, dyslipidemia, and diabetes utilized laboratory data in addition to that information.

### 2.3 Endpoints of the Study Outcomes

The objective of this study was to determine newly developed LROs, including hepatocellular carcinoma (HCC) and liver decompensation‐related complications, including ascites, variceal bleeding, or hepatic encephalopathy, after achieving an SVR. The diagnosis of LRO was based on the International Classification of Diseases, Ninth Revision, Clinical Modification (ICD‐9‐CM) or Tenth Revision (ICD‐10) on the catastrophic illness or cancer registry databases of the Taiwan National Insurance Database (Table S1). Mortality or liver transplantation before major events was adjusted as a competing risk. The follow‐up period was calculated from the time point of SVR to the occurrence of new‐onset major events, death, or transplantation or until December 31, 2019, whichever came first.

### 2.4 Statistical Analysis

Continuous variables are reported as the means ± standard deviations, whereas categorical variables are presented as numbers (percentages). Chi‐square or Fisher's exact tests were used for categorical comparisons, and Student's *t‐*test was used for continuous variables. A 1:1 propensity score matching for age, sex, FIB‐4, and eGFR was performed between SLD and non‐SLD patients to further compare the incidence of LROs. Kaplan–Meier analysis, modified using Gray's cumulative incidence method, was used to compare incidences between groups. The Cox subdistribution hazards model was used to identify independent risk factors associated with LRO. All the statistical analyses were performed using the SAS Enterprise Guide (version 9.4, SAS Institute Inc., Cary, NC, USA), with a two‐sided *p* value of < 0.05 indicating statistical significance.

## 3 Results

### 3.1 Characteristics of CHC Patients With or Without SLD

**FIGURE 1:**
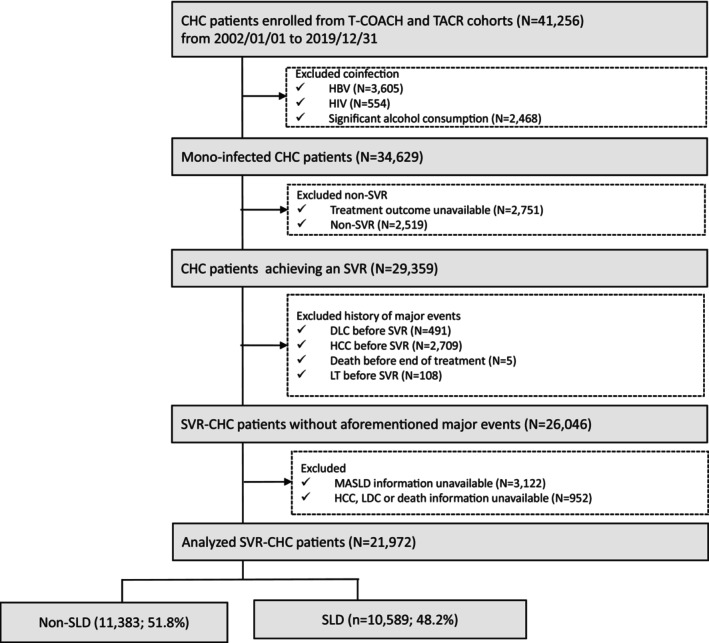
Patient flowchart.

A total of 21,972 CHC patients were enrolled for analysis (Figure 1). The mean age was 60.0 years, and males accounted for 43.1% of the population. Among them, 10,589 (48.2%) patients had SLD (4371 patients used sonography, and the rest were defined by HSI), of whom 10,066 (95.1%) carried at least one CMRF and were viewed as having MASLD. Compared with patients without SLD, those with SLD were younger, had a higher BMI, and had higher proportions of male sex, diabetes, hypertension, dyslipidemia, and a history of cardiocerebral vascular diseases. With respect to laboratory data, patients with SLD had higher levels of liver enzymes, eGFR, and platelet counts and had more unfavorable lipid and sugar profiles. With respect to liver disease severity, patients with SLD had a lower FIB‐4 score and a smaller proportion of liver cirrhosis (Table 1).

**TABLE 1:** Demographic data of SLD and non‐SLD patients.

	Total	Non‐SLD	SLD	*p*
No. of patients	21,972	11,383	10,589	
Age (years)	60.0 ± 12.6	62.0 ± 12.6	57.8 ± 12.2	< 0.001
≤ 65	14,338 (65.3)	6689 (58.8)	7649 (72.2)	< 0.001
> 65	7634 (34.7)	4694 (41.2)	2940 (25.2)	
Gender				
Male	9472 (43.1)	4777 (42.0)	4695 (44.3)	< 0.001
Female	12,500 (56.9)	6606 (58.0)	5894 (55.7)	
Body mass index (kg/m^2^)	24.6 ± 3.5	23.1 ± 2.6	26.3 ± 3.5	< 0.001
≥ 23	16,190 (73.7)	6726 (59.1)	9464 (89.4)	< 0.001
≥ 27	4235 (19.3)	514 (4.5)	3721 (35.1)	< 0.001
Diabetes				
No	12,853 (61.6)	7488 (69.2)	5365 (53.4)	< 0.001
Prediabetes	3888 (18.6)	1961 (18.1)	1927 (19.2)	
Yes	4138 (19.8)	1380 (12.7)	2758 (27.4)	
Hypertension	5736 (28.8)	2688 (25.7)	3048 (32.1)	< 0.001
Dyslipidemia	2256 (11.3)	951 (9.1)	1305 (13.8)	< 0.001
Chronic kidney disease	2332 (15.7)	1413 (17.0)	919 (14.0)	< 0.001
Cerebrovascular disease	404 (2.7)	262 (3.1)	142 (2.2)	< 0.001
Cardiovascular disease	1412 (9.5)	768 (9.2)	644 (9.8)	0.228
Liver cirrhosis				
No	16,977 (77.3)	8616 (75.7)	8361 (79.0)	< 0.001
Yes	4995 (22.7)	2767 (24.3)	2228 (21.0)	
CMRF				
No	2775 (12.6)	2252 (29.8)	523 (4.9)	< 0.001
Yes	19,197 (87.4)	9131 (80.2)	10,066 (95.1)	
Number: 1	9267 (42.2)	5116 (44.9)	4151 (39.2)	< 0.001
Numbers: 2	4828 (22.0)	2285 (20.1)	2543 (24.0)	
Numbers: ≥ 3	5102 (23.2)	1730 (15.2)	3372 (31.8)	
MASLD				
No	11,906 (54.2)	11,383 (100.0)	523 (4.9)	—
Yes	10,066 (45.8)	0 (0.0)	10,066 (95.1)	
Fasting glucose (mg/dL)	108.7 ± 36.2	103.5 ± 30.9	113.5 ± 39.9	< 0.001
HbA1c (%)	6.0 ± 1.2	5.8 ± 1.0	6.3 ± 1.3	< 0.001
Total cholesterol (mg/dL)	171.2 ± 35.4	170.9 ± 36.0	171.5 ± 34.8	0.422
Triglyceride (mg/dL)	104.6 ± 66.2	97.2 ± 56.0	111.3 ± 73.7	< 0.001
≥ 150	2636 (12.8)	1041 (9.7)	1595 (16.1)	< 0.001
HDL‐C (mg/dL)	49.3 ± 15.5	51.5 ± 16.1	47.2 ± 14.6	
HDL‐C				
< 40 for male or < 50 for female	4694 (23.0)	1980 (18.6)	2714 (27.8)	< 0.001
LDL‐C (mg/dL)	100.6 ± 30.4	99.1 ± 30.9	102.1 ± 29.9	
AFP (ng/mL)	11.9 ± 132.0	13.3 ± 176.3	10.5 ± 49.7	0.122
AST (U/L)	70.0 ± 57.2	66.1 ± 58.0	74.2 ± 56.1	< 0.001
≤ 80	15,734 (71.6)	8495 (74.6)	7239 (68.4)	< 0.001
> 80	6238 (28.4)	2888 (25.4)	3350 (31.6)	
ALT (U/L)	95.4 ± 93.4	75.7 ± 77.6	116.7 ± 103.7	< 0.001
≤ 80	12,823 (58.4)	7943 (69.8)	4880 (46.1)	< 0.001
> 80	9149 (41.6)	3440 (30.2)	5709 (53.9)	
Platelet counts (×10^3^/μL)	177.7 ± 63.5	174.0 ± 64.7	181.7 ± 62.0	< 0.001
Creatinine (mg/dL)	1.10 ± 1.45	1.18 ± 1.63	1.02 ± 1.23	< 0.001
eGFR (mL/min/1.73 m^2^)	88.0 ± 29.9	86.4 ± 31.4	89.6 ± 28.2	< 0.001
≥ 60	19,342 (88.0)	9787 (86.0)	9555 (90.2)	< 0.001
< 60	2630 (12.0)	1596 (14.0)	1034 (9.8)	
FIB‐4	3.08 ± 2.76	3.45 ± 3.08	2.69 ± 2.32	< 0.001
≤ 3.25	14,960 (68.1)	7035 (61.8)	7925 (74.8)	< 0.001
> 3.25	7012 (31.9)	4348 (38.2)	2664 (25.2)	
Antiviral agent				
DAA	14,887 (67.8)	8323 (73.1)	6564 (62.0)	< 0.001
IFN	7085 (32.2)	3060 (26.9)	4025 (38.0)	
Liver‐related outcomes				
Person‐years	71,000	30,983	40,017	
Follow‐up (years)				
Mean ± SD	3.2 ± 3.8	2.7 ± 3.4	3.8 ± 4.1	< 0.001
Median (Q1–Q3)	1.5 (0.5–5.4)	1.3 (0.4–3.3)	1.8 (0.5–6.9)	
No. (%)	745 (3.4)	422 (3.7)	323 (3.1)	< 0.001
Annual incidence (per 10,000 PYs)	104.9	136.2	80.7	< 0.001
Liver decompensation				
Person‐years	72,811	31,965	40,846	
Follow‐up (years)				
Mean ± SD	3.3 ± 3.8	2.8 ± 3.5	3.9 ± 4.1	< 0.001
Median (Q1–Q3)	1.5 (0.5–5.6)	1.4 (0.4–3.6)	1.9 (0.5–7.2)	
No. (%)	53 (0.2)	35 (0.3)	18 (0.2)	< 0.001
Annual incidence (per 10,000 PYs)	7.3	10.9	4.4	0.001
HCC				
Person‐years	71,104	31,048	40,055	
Follow‐up (years)				
Mean ± SD	3.2 ± 3.8	2.7 ± 3.4	3.8 ± 4.1	< 0.001
Median (Q1–Q3)	1.5 (0.5–5.4)	1.4 (0.4–3.3)	1.8 (0.5–6.9)	
No. (%)	702 (3.2)	395 (3.5)	307 (2.9)	< 0.001
Annual incidence (per 10,000 PYs)	98.7	124.0	76.6	< 0.001

Abbreviations: AFP, alpha fetoprotein; ALT, alanine aminotransferase; AST, aspartate aminotransferase; CMRFs, cardiometabolic risk factors; DAA, directly acting antiviral agent; eGFR, estimated glomerular filtration rate; FIB‐4, fibrosis‐4 index; HbA1c, glycated hemoglobin; HCC, hepatocellular carcinoma; HDL‐C, high density lipoprotein cholesterol; IFN, interferon‐based therapy; LDL‐C, low density lipoprotein cholesterol; MASLD, metabolic dysfunction‐associated steatotic liver disease; PYs, person‐years; SLD, steatotic liver disease.

### 3.2 The Incidence of Liver‐Related Outcomes

During a follow‐up period of 71,000 person‐years (PYs), 745 (3.4%) patients developed LROs (annual incidence 104.9 per 10,000 PYs), including 702 (3.2%) patients with HCC and 53 (0.2%) patients with liver decompensation. The annual incidences of liver decompensation (10.9 vs. 4.4 per 10,000 PYs, *p* < 0.001), HCC (124.0 vs. 76.6 per 10,000 PYs, *p* < 0.001), and LRO (136.2 vs. 80.7 per 10,000 PYs, *p* < 0.001) were significantly greater in patients without SLD than in those with SLD (Table 1).

### 3.3 Risk Factors Associated With LROs

**FIGURE 2:**
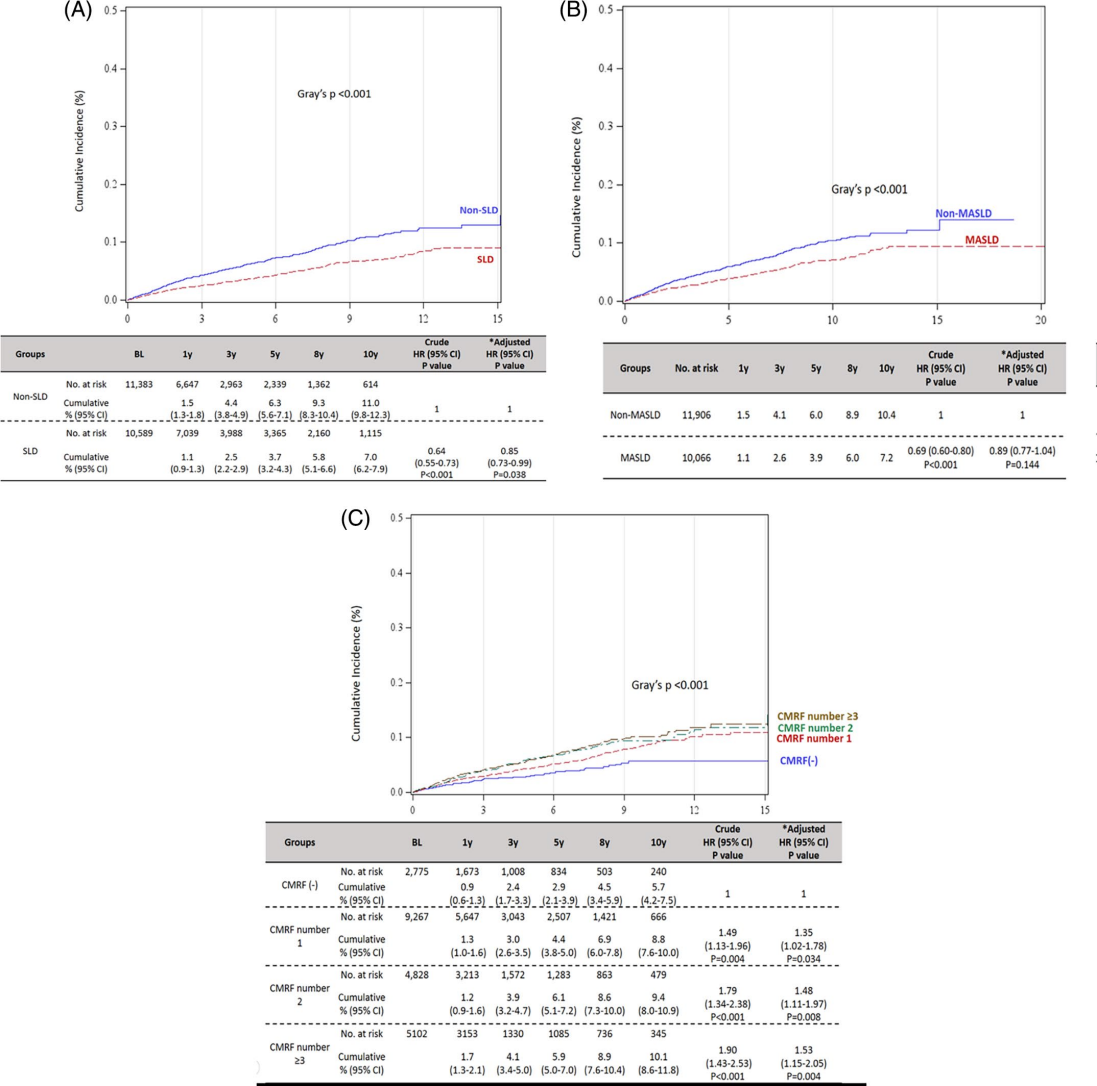
(A) Cumulative incidence of liver‐related outcomes between patients with or without steatotic liver disease. (B) Cumulative incidence of liver‐related outcomes between patients with or without metabolic dysfunction‐associated steatotic liver disease. (C) Cumulative incidence of liver‐related outcomes among patients with different numbers of CMRFs. *Adjusted for age > 65 vs. ≤ 65 years, eGFR ≥ 60 mL/min/1.73 m^2^ versus < 60 mL/min/1.73 m^2^ and FIB‐4 ≤ 3.25 or > 3.25. CMRF, cardiometabolic risk factor; MASLD, metabolic dysfunction‐associated steatotic liver disease; SLD, steatotic liver disease.

Compared with patients without LROs, those with LRO development were older; had higher AST, ALT, and FIB‐4 levels; had a greater proportion of liver cirrhosis and chronic kidney disease; and had more CMRFs (Table 2). The 1‐, 3‐, and 5‐year cumulative incidence rates of LROs were 1.1%, 2.5%, and 3.7%, respectively, for patients with SLD, which were lower than the 1.5%, 4.4%, and 6.3%, respectively, for patients without SLD (Gray's *p* < 0.001). Cox regression analysis revealed that SLD was independently associated with a lower risk of LRO (adjusted hazard ratio [aHR]/95% confidence interval [CI]: 0.85/0.73–0.99, *p* = 0.038) (Table 2 and Figure 2). The 1‐, 3‐, and 5‐year cumulative incidence rates of LRO were 1.1%, 2.6%, and 3.9%, respectively, for patients with MASLD, which were lower than the 1.5%, 4.1%, and 6.0%, respectively, for patients without MASLD (Gray's *p* < 0.001). However, multivariate analysis did not reveal a significant difference in LRO risk between patients with and without MASLD (Table 2 and Figure 2B). On the other hand, there was an increasing trend toward increased LRO risk in patients who carried more CMRFs than in those without any CMRF (aHR/CI: 1.35/1.02–1.78, 1.48/1.11–1.97, and 1.53/1.15–2.05 for 1, 2, and ≥ 3 CMRFs, respectively) (Table 2 and Figure 2C).

**TABLE 2:** Risk factors for liver‐related outcomes in all patients.

		*N*	LRO, *n* (%)	Crude HR (95% CI)	*p*	Adjusted HR (95% CI)^a^	*p*
Age (years)	≤ 65	14,338	433 (3.0)	1		1	
	> 65	7634	312 (4.1)	2.37 (2.04–2.74)	< 0.001	1.40 (1.18–1.66)	< 0.001
Gender	Male	9472	400 (4.2)	1		1	
	Female	12,500	345 (2.8)	0.80 (0.70–0.93)	0.003	0.64 (0.55–0.74)	< 0.001
AST (IU/L)	≤ 80	15,734	341 (2.2)	1			
	> 80	6238	404 (6.5)	1.89 (1.64–2.19)	< 0.001		
ALT (IU/L)	≤ 80	12,823	281 (2.2)	1			
	> 80	9149	464 (5.1)	1.20 (1.03–1.39)	0.020		
eGFR (mL/min/1.73 m^2^)	≥ 60	19,342	645 (3.3)	1		1	
	< 60	2630	100 (3.8)	1.65 (1.34–2.04)	< 0.001	1.23 (0.98–1.53)	0.072
FIB‐4	≤ 3.25	14,960	243 (1.6)	1		1	
	> 3.25	7012	502 (7.2)	4.45 (3.82–5.19)	< 0.001	4.07 (3.43–4.83)	< 0.001
SLD^a^	−	11,383	422 (3.7)	1		1	
	+	10,589	323 (3.1)	0.64 (0.55–0.73)	< 0.001	0.85 (0.73–0.99)	0.038
CMRF^a^	−	2775	62 (2.2)	1		1	
	+	19,197	683 (3.6)	1.67 (1.29–2.17)	< 0.001		
	Number 1	9267	294 (3.2)	1.49 (1.13–1.96)	0.004	1.35 (1.02–1.78)	0.034
	Numbers 2	4828	195 (4.0)	1.79 (1.34–2.38)	< 0.001	1.48 (1.11–1.97)	0.008
	Numbers ≥ 3	5102	194 (3.8)	1.90 (1.43–2.53)	< 0.001	1.53 (1.15–2.05)	0.004
SLD/CMRF^a^	SLD(−)/CMRF(−)	2252	50 (2.2)	1.71 (0.91–3.20)	0.093	1.33 (0.71–2.47)	0.377
	SLD(−)/CMRF(+)	9131	372 (4.1)	3.31 (1.87–5.86)	< 0.001	1.97 (1.11–3.50)	0.002
	SLD(+)/CMRF(−)	523	12 (2.3)	1		1	
	SLD(+)/CMRF(+)	10,066	311 (3.1)	1.96 (1.10–3.47)	0.022	1.62 (0.91–2.86)	0.099
MASLD^a^	No	11,906	434 (3.7)	1		1	
	Yes	10,066	311 (3.1)	0.69 (0.60–0.80)	< 0.001	0.89 (0.77–1.04)	0.144
CMRF−^a^		2775	62 (2.2)	1		1	
CMRF(+, number 1)/SLD(−)		5116	195 (3.8)	2.02 (1.52–2.69)	< 0.001	1.57 (1.17–2.09)	0.002
CMRF(+, numbers ≥ 2)/SLD(−)		4015	177 (4.4)	2.44 (1.82–3.26)	< 0.001	1.61 (1.20–2.17)	0.002
CMRF(+, number 1)/SLD(+)		4151	99 (2.4)	0.98 (0.72–1.35)	0.914	1.07 (0.77–1.47)	0.695
CMRF(+, number ≥ 2)/SLD(+)		5915	212 (3.6)	1.53 (1.15–2.04)	0.003	1.44 (1.09–1.92)	0.011

Abbreviations: ALT, alanine aminotransferase; AST, aspartate aminotransferase; CI, confidence intervals; CMRFs, cardiometabolic risk factors; eGFR, estimated glomerular filtration rate; FIB‐4, fibrosis‐4 index; HR, hazard ratio; MASLD, metabolic dysfunction‐associated steatotic liver disease; SLD, steatotic liver disease.

^a^ The individual covariates regarding SLD, MASLD, and CMRF were put into cox‐regression analysis by adjusting age, sex, FIB‐4 value of 3.25 and eGFR of 60 mL/min/1.73 m^2^ in separate models.

### 3.4 Risk of LRO Stratified by SLD Status and the Presence of CMRFs

**FIGURE 3:**
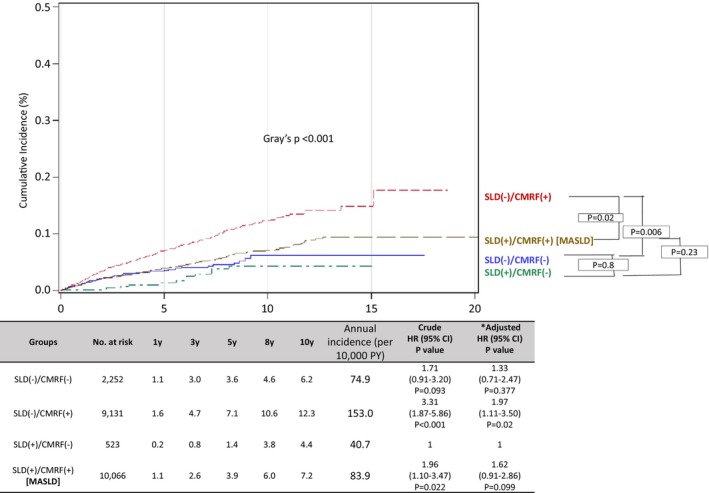
Cumulative incidence of liver‐related outcomes in patients stratified by SLD status and the presence of CMRFs. *Adjusted for age > 65 versus ≤ 65 years, eGFR ≥ 60 mL/min/1.73 m^2^ versus < 60 mL/min/1.73 m^2^ and FIB‐4 ≤ 3.25 or > 3.25. CMRF, cardiometabolic risk factor; SLD, steatotic liver disease.

We further analyzed the risk of LRO by incorporating SLD status and the presence of CMRFs. The 5‐year risk was lowest in SLD+/CMRF− patients (1.4%) and highest in SLD−/CMRF+ patients (7.1%). Cox regression analysis revealed that patients without SLD who carried CMRFs were independently associated with a high risk of LRO compared with SLD patients without any CMRF carriage (simple steatosis) (aHR/CI: 1.97/1.11–3.50, *p* = 0.02) (Table 2 and Figure 3). Compared with subjects without any CMRF, SLD patients with at least two CMRFs (aHR/CI: 1.44/1.09–1.92, *p* = 0.011) and non‐SLD patients with any CMRF (aHR/CI: 1.57/1.17–2.09, *p* = 0.002 for 1 CMRF; aHR/CI: 1.61/1.20–2.17, *p* = 0.002 for ≥ 2 CMRFs) had a significant risk of LROs (Table 2).

### 3.5 Role of SLD and CMRF Burden in Patients With or Without Liver Cirrhosis

Compared with their counterparts, neither the presence of SLD/MASLD nor CMRF was associated with LRO in cirrhotic patients. Among noncirrhotic patients, when patients without CMRF were used as the reference, the risk of LROs did not differ significantly among SLD patients regardless of CMRF carriage, whereas the risk increased significantly among non‐SLD patients with one CMRF (aHR/CI: 1.53/1.05–2.22, *p* = 0.028) or more than two CMRFs (aHR/CI: 1.60/1.09–2.36, *p* = 0.017) (Table 3).

**TABLE 3:** Risk factors for liver‐related outcomes stratified by cirrhotic status.

			*N*	Incidence, *n* (%)	cHR (95% CI)	*p*	aHR (95% CI)^a^	*p*
Non‐LC	SLD^a^	−	8616	219 (2.5)	1		1	
		+	8361	203 (2.4)	0.67 (0.55–0.81)	< 0.001	0.85 (0.70–1.03)	0.099
	CMRF^a^	−	2328	38 (1.6)	1		1	
		+	14,649	384 (2.6)	1.64 (1.18–2.29)	0.004	1.38 (0.99–1.94)	0.058
		Number 1	7520	171 (2.3)	1.47 (1.03–2.09)	0.032	1.30 (0.91–1.85)	0.150
		Numbers 2	3550	116 (3.3)	1.86 (1.29–2.68)	0.001	1.51 (1.05–2.19)	0.027
		Numbers ≥ 3	3579	97 (2.7)	1.76 (1.21–2.56)	0.003	1.41 (0.97–2.06)	0.076
	SLD/CMRF^a^	SLD(−)/CMRF(−)	1852	29 (1.6)	1.52 (0.72–3.21)	0.272	1.21 (0.57–2.55)	0.625
		SLD(−)/CMRF(+)	6764	190 (2.8)	2.91 (1.49–5.68)	0.002	1.79 (0.91–3.51)	0.092
		SLD(+)/CMRF(−)	476	9 (1.9)	1		1	
		SLD(+)/CMRF(+) [MASLD]	7885	194 (2.5)	1.80 (0.93–3.52)	0.084	1.45 (0.74–2.83)	0.281
		Number 1	3548	69 (1.9)	1.45 (0.73–2.91)	0.292	1.23 (0.61–2.48)	0.568
		Numbers 2	1954	63 (3.2)	2.12 (1.06–4.26)	0.035	1.65 (0.82–3.33)	0.160
		Numbers ≥ 3	2383	62 (2.6)	2.03 (1.01–4.09)	0.047	1.48 (0.73–3.01)	0.275
	MASLD^a^	−	9092	228 (2.5)	1		1	
		+	7885	194 (2.5)	0.74 (0.61–0.90)	0.002	0.79 (0.65–0.96)	0.016
	CMRF(−)^a^		2328	38 (1.6)	1		1	
	CMRF(+, number 1)/SLD(−)		3972	102 (2.6)	1.97 (1.35–2.86)	< 0.001	1.53 (1.05–2.22)	0.028
	CMRF(+, numbers ≥ 2)/SLD(−)		2792	88 (3.2)	2.41 (1.65–3.53)	< 0.001	1.60 (1.09–2.36)	0.017
	CMRF(+, number 1)/SLD(+)		3548	69 (1.9)	1.07 (0.72–1.60)	0.725	1.07 (0.72–1.60)	0.740
	CMRF(+, numbers ≥ )/SLD(+)		4337	125 (2.9)	1.54 (1.07–2.21)	0.020	1.40 (0.97–2.01)	0.073
LC	SLD^a^	−	2767	203 (7.3)	1		1	
		+	2228	120 (5.4)	0.81 (0.64–1.01)	0.061	0.94 (0.75–1.19)	0.622
	CMRF^a^	−	447	24 (5.4)	1		1	
		+	4548	299 (6.6)	1.39 (0.91–2.11)	0.13	1.35 (0.88–2.06)	0.171
		Number 1	1747	123 (7.0)	1.35 (0.87–2.10)	0.184	1.33 (0.85–2.07)	0.211
		Numbers 2	1278	79 (6.2)	1.34 (0.84–2.13)	0.215	1.29 (0.81–2.06)	0.281
		Numbers ≥ 3	1523	97 (6.4)	1.48 (0.94–2.33)	0.092	1.43 (0.90–2.26)	0.132
	SLD/CMRF^a^	SLD(−)/CMRF(−)	400	21 (5.3)	1.27 (0.40–4.03)	0.68	1.45 (0.47–4.44)	0.514
		SLD(−)/CMRF(+)	2367	182 (7.7)	1.90 (0.65–5.56)	0.244	1.91 (0.67–5.43)	0.228
		SLD(+)/CMRF(−)	47	3 (6.4)	1		1	
		SLD(+)/CMRF(+) [MASLD]	2181	117 (5.4)	1.47 (0.50–4.34)	0.483	1.77 (0.62–5.07)	0.285
		Number 1	603	30 (5.0)	1.33 (0.44–4.05)	0.617	1.65 (0.55–4.93)	0.372
		Numbers 2	589	26 (4.4)	1.27 (0.42–3.92)	0.672	1.49 (0.49–4.53)	0.480
		Numbers ≥ 3	989	61 (6.2)	1.90 (0.64–5.61)	0.246	2.01 (0.69–5.85)	0.201
	MASLD^a^	−	2814	206 (7.3)	1		1	
		+	2181	117 (5.4)	0.83 (0.66–1.04)	0.096	0.97 (0.77–1.23)	0.824
	CMRF(−)^a^		447	24 (5.4)	1		1	1
	CMRF(+, number 1)/SLD(−)		1144	93 (8.1)	1.50 (0.95–2.35)	0.081	1.39 (0.88–2.19)	0.155
	CMRF(+, numbers ≥ 2)/SLD(−)		1223	89 (7.3)	1.59 (1.01–2.50)	0.048	1.38 (0.87–2.20)	0.172
	CMRF(+, number 1)/SLD(+)		603	30 (5.0)	1.03 (0.60–1.77)	0.913	1.16 (0.68–2.00)	0.586
	CMRF(+, numbers ≥ 2)/SLD(+)		1578	87 (5.5)	1.27 (0.80–2.00)	0.314	1.34 (0.85–2.13)	0.213

Abbreviations: CI, confidence intervals; CMRFs, cardiometabolic risk factors; HR, hazard ratio; MASLD, metabolic dysfunction‐associated steatotic liver disease; SLD, steatotic liver disease.

^a^ The individual covariates regarding SLD, MASLD, and CMRF were put into cox‐regression analysis by adjusting age, sex, FIB‐4 value of 3.25, and eGFR of 60 mL/min/1.73 m^2^ in separate models.

### 3.6 Risk of LRO Between SLD and Non‐SLD Patients After Propensity Score Matching

As shown in the Table S2, a total of 18,412 patients (9206 in SLD and non‐SLD group, respectively) were enrolled after propensity score matching. Compared to SLD patients, those without SLD continued to have a higher incidence of LROs (114.5 vs. 90.2 per 10,000 PYs, *p* = 0.003), including liver decompensation (9.9 vs. 4.3 per 10,000 PYs, *p* = 0.003, *p* = 0.008) and HCC (106.1 vs. 86.0 per 10,000 PYs, *p* = 0.012). Similarly, by stratifying SLD status and the presence of CMRFs, the 5‐year risk of LRO was lowest in SLD+/CMRF− patients (1.7%) and highest in SLD‐/CMRF+ patients (6.1%) (HR/CI: 2.52/1.38–4.59, *p* = 0.003) (Figure S2).

## 4 Discussion

In this nationwide study, we demonstrated that the annual incidence of LROs, including HCC and liver decompensation, was 1.05% after HCV eradication in CHC patients. In general, non‐SLD patients had a greater risk of LROs than those with SLD did. When CMRFs were taken into consideration, there was a dose–response effect of the number of CMRFs in terms of LRO risk. Overall, non‐SLD patients who carried CMRFs were at the greatest risk of LRO compared to their counterpart patients. Furthermore, the impact of the CMRF burden on LRO was particularly enhanced in noncirrhotic patients.

The presence of hepatic steatosis has been reported to be associated with increased HCC risk in CHC patients after achieving an SVR. For example, a cohort enrolling 699 IFN‐treated patients revealed that patients with hepatic steatosis had a 2.1‐fold greater risk of HCC development than did those without hepatic steatosis [29]. Liu et al. enrolled 1598 DAA‐treated CHC patients who achieved an SVR. After mediating the effect of underlying CMRFs, MASLD independently increased HCC risk compared with non‐SLD patients [30]. In contrast, a study enrolling 2611 DAA‐treated patients who had advanced liver fibrosis revealed that hepatic steatosis was no longer associated with HCC occurrence if the factor of metabolic dysfunction was adjusted [17]. We observed an inverse association of hepatic steatosis with HCC and liver decompensation after achieving an SVR in this population‐based study. One of the critical explanations was that non‐SLD patients were older and had more advanced liver disease than did SLD patients. Hepatic steatosis may trigger liver fibrosis in the early stage of liver disease, but it is inversely associated with liver fibrosis due to the burnout phenomenon in the late course of liver disease [22]. Coincidentally, we have shown that there is a bidirectional trajectory in terms of the existence of hepatic steatosis from fibrotic Stage 0 (F0) to Stage 4 (F4) in a biopsy‐proven cohort comprising 1120 CHC patients. The proportion of hepatic steatosis increased with the progression of the fibrotic stage from F0 (20%), F1 (40%) to F2 (63%) but started to decrease from F3 (55%) to F4 (41%) [31]. Over time, the presence of hepatic steatosis becomes a time‐dependent and uncontrolled variable for liver fibrosis and greatly confounds the interpretation of its association with LRO. For example, in Liu's study, the mean age of the patients was younger than that in the current study, the mean liver stiffness was only 6.1 kPa by transient elastography, and patients without MASLD had less advanced liver fibrosis than did patients with MASLD [30]. The inverse characteristics of patients at enrollment may attribute to the discrepant interpretations of the impact of hepatic steatosis on long‐term outcomes. Different modalities in the diagnosis of SLD (e.g., elastography, ultrasonography, and HSI) may also in part account for the discordance.

Metabolic dysfunction is one of the triggers of hepatic steatosis and may confound HCC risk. Beyond the issue of hepatic steatosis, metabolic disarrangement, such as diabetes, might be more critical for predicting the severity and outcome of liver disease [11, 32]. For instance, we have denoted that DM and obesity are the two risk factors in 7249 CHC patients with curative antivirals [25]. We further showed that even a subclinical diabetic status might increase the risk of HCC after HCV eradication [33]. Another CMRF, an increased body mass index, has also been shown to be a risk factor for LROs in both HCV monoinfected and HBV dual‐infected patients who achieve an SVR [34, 35]. Recently, a Korean cohort study revealed that CMRFs were associated with deferred fibrosis regression and an increased risk of liver decompensation and HCC after HCV eradication [36]. In the present study, we further noted that the patient's risk of LROs increased in parallel with the number of CMRFs. This finding was in line with a large HBV‐suppressed cohort, which showed that having ≥ 2 unfavorable metabolic components was associated with a significantly increased risk of cirrhosis development [37]. Moreover, we demonstrated that the influence of the CMRF burden on unfavorable liver outcomes was noticeable only in noncirrhotic patients. These results also echo those of previous reports showing that glucose abnormalities are a risk factor for post‐SVR‐HCC, particularly in patients without advanced liver fibrosis [33, 38].

The current study was limited by the failure to use liver biopsy or quantitative image modalities such as elastography or MRI‐PDFF as the reference for the diagnosis of hepatic steatosis. Rather, we used ultrasonography and a non‐invasive biomarker to define SLD in the two nationwide cohorts, which may lead to misclassification bias. Furthermore, the definition of liver decompensation was defined by the catastrophic illness registry, in which the severity and clinical course could not be judged individually from a registry‐based study. We also failed to adopt posttreatment status as a link to the outcomes. We recently demonstrated that the proportion of SLD and CMRF presence did not change 6 months after HCV eradication, nor did the proportion of MASLD [10]. A longer follow‐up period to address the linkage between the evolution of SLD, CMRFs and LROs is warranted. In conclusion, liver‐related events continue to occur in a subset of CHC patients after HCV eradication, and the presence of cardiometabolic factors is a critical determinant of LROs, particularly in noncirrhotic patients. The holistic management of metabolic disarrangement should be adopted beyond HCC surveillance in the post‐SVR era. Further studies are needed to validate the findings in areas with different geographic and ethnic backgrounds.

## Conflicts of Interest

The authors declare no conflicts of interest.

## Supporting information


**Figure S1:** Cumulative incidence liver‐related outcomes in patients with different CMRF burden and SLD status stratified by cirrhotic status. CMRF, cardiometabolic risk factor; SLD, steatotic liver disease.
**Figure S2:** Cumulative incidence of liver‐related outcomes in patients stratified by SLD status and the presence of CMRFs after propensity score matching. CMRF, cardiometabolic risk factor; SLD, steatotic liver disease.

**Table S1:** Disease code (ICD‐9‐CM and ICD‐10) for major outcomes and competing risk.
**Table S2:** Baseline characteristics and incidence of liver related outcomes of SLD and non‐SLD patients before and after propensity score matching (PSM).

## Data Availability

The data that support the findings of this study are available on request from the corresponding author. The data are not publicly available due to privacy or ethical restrictions.
