# Supplementary material for: Impact of Cardiometabolic Risk Factors and Steatotic Liver Disease on Liver‐Related Outcomes in Patients With Chronic Hepatitis C After Curative Antiviral Therapy
Source: Kaohsiung J Med Sci. 2026 Apr 22:e70214. Online ahead of print. doi: 10.1002/kjm2.70214 (PMC13399790; doi:10.1002/kjm2.70214)
Supplement: Supplementary file 1 — Figure S1: Cumulative incidence liver‐related outcomes in patients with different CMRF burden and SLD status stratified by cirrhotic status. CMRF, cardiometabolic risk factor; SLD, steatotic liver disease. Figure S2: Cumulative incidence of liver‐related outcomes in patients stratified by SLD status and the presence of CMRFs after propensity score matching. CMRF, cardiometabolic risk factor; SLD, steatotic liver disease. [file KJM2-9999-e70214-s002.pptx]

## Slide 1
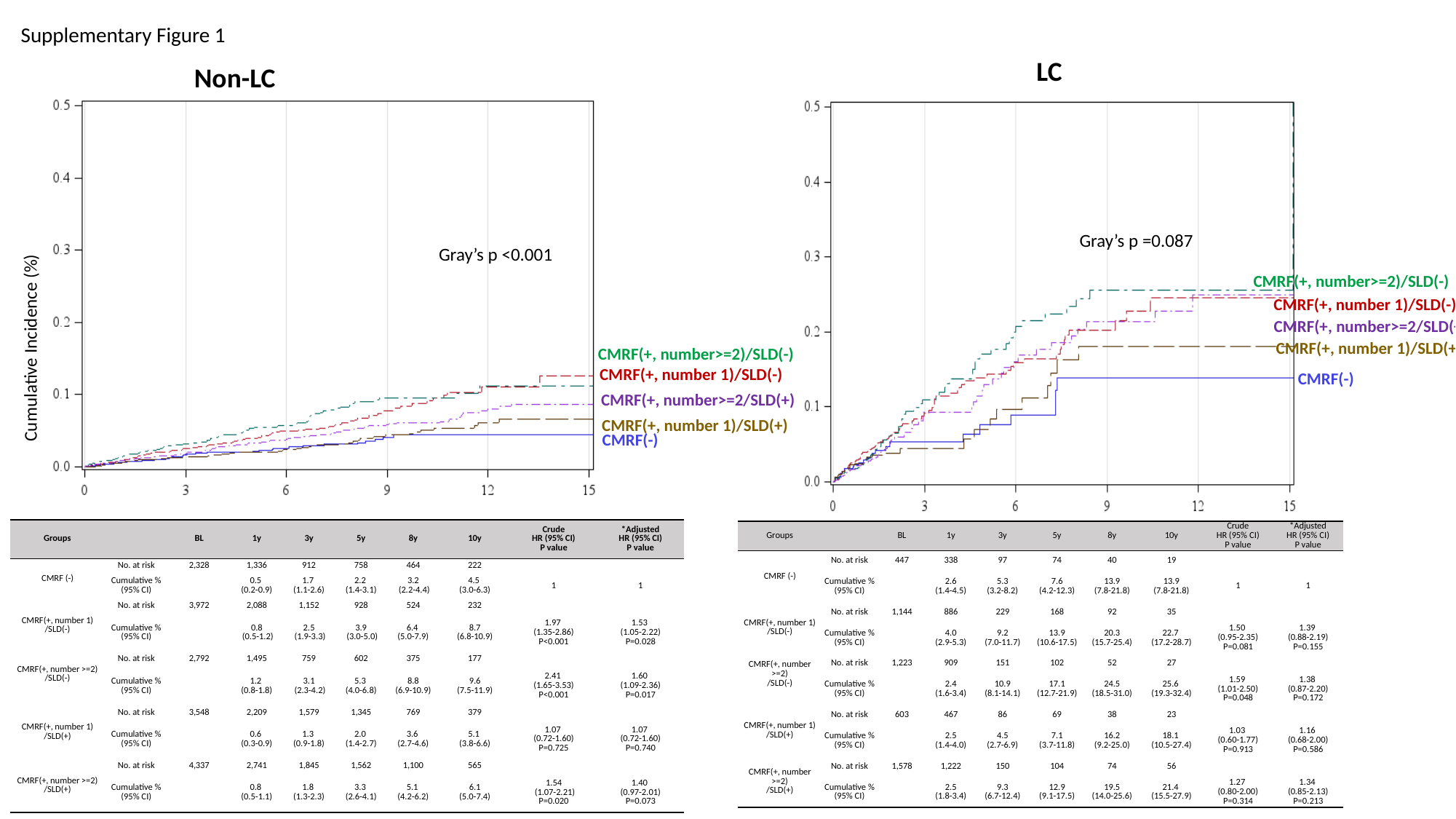

Supplementary Figure 1
LC
Non-LC
Gray’s p =0.087
Gray’s p <0.001
Cumulative Incidence (%)
CMRF(+, number>=2)/SLD(-)
CMRF(+, number 1)/SLD(-)
CMRF(+, number>=2/SLD(+)
CMRF(+, number 1)/SLD(+)
CMRF(+, number>=2)/SLD(-)
CMRF(+, number 1)/SLD(-)
CMRF(-)
CMRF(+, number>=2/SLD(+)
CMRF(+, number 1)/SLD(+)
CMRF(-)
| Groups | | BL | 1y | 3y | 5y | 8y | 10y | Crude HR (95% CI) P value | \*Adjusted HR (95% CI) P value |
| --- | --- | --- | --- | --- | --- | --- | --- | --- | --- |
| CMRF (-) | No. at risk | 2,328 | 1,336 | 912 | 758 | 464 | 222 | | |
| | Cumulative % (95% CI) | | 0.5 (0.2-0.9) | 1.7 (1.1-2.6) | 2.2 (1.4-3.1) | 3.2 (2.2-4.4) | 4.5 (3.0-6.3) | 1 | 1 |
| CMRF(+, number 1) /SLD(-) | No. at risk | 3,972 | 2,088 | 1,152 | 928 | 524 | 232 | | |
| | Cumulative % (95% CI) | | 0.8 (0.5-1.2) | 2.5 (1.9-3.3) | 3.9 (3.0-5.0) | 6.4 (5.0-7.9) | 8.7 (6.8-10.9) | 1.97 (1.35-2.86) P<0.001 | 1.53 (1.05-2.22) P=0.028 |
| CMRF(+, number >=2) /SLD(-) | No. at risk | 2,792 | 1,495 | 759 | 602 | 375 | 177 | | |
| | Cumulative % (95% CI) | | 1.2 (0.8-1.8) | 3.1 (2.3-4.2) | 5.3 (4.0-6.8) | 8.8 (6.9-10.9) | 9.6 (7.5-11.9) | 2.41 (1.65-3.53) P<0.001 | 1.60 (1.09-2.36) P=0.017 |
| CMRF(+, number 1) /SLD(+) | No. at risk | 3,548 | 2,209 | 1,579 | 1,345 | 769 | 379 | | |
| | Cumulative % (95% CI) | | 0.6 (0.3-0.9) | 1.3 (0.9-1.8) | 2.0 (1.4-2.7) | 3.6 (2.7-4.6) | 5.1 (3.8-6.6) | 1.07 (0.72-1.60) P=0.725 | 1.07 (0.72-1.60) P=0.740 |
| CMRF(+, number >=2) /SLD(+) | No. at risk | 4,337 | 2,741 | 1,845 | 1,562 | 1,100 | 565 | | |
| | Cumulative % (95% CI) | | 0.8 (0.5-1.1) | 1.8 (1.3-2.3) | 3.3 (2.6-4.1) | 5.1 (4.2-6.2) | 6.1 (5.0-7.4) | 1.54 (1.07-2.21) P=0.020 | 1.40 (0.97-2.01) P=0.073 |
| Groups | | BL | 1y | 3y | 5y | 8y | 10y | Crude HR (95% CI) P value | \*Adjusted HR (95% CI) P value |
| --- | --- | --- | --- | --- | --- | --- | --- | --- | --- |
| CMRF (-) | No. at risk | 447 | 338 | 97 | 74 | 40 | 19 | | |
| | Cumulative % (95% CI) | | 2.6 (1.4-4.5) | 5.3 (3.2-8.2) | 7.6 (4.2-12.3) | 13.9 (7.8-21.8) | 13.9 (7.8-21.8) | 1 | 1 |
| CMRF(+, number 1) /SLD(-) | No. at risk | 1,144 | 886 | 229 | 168 | 92 | 35 | | |
| | Cumulative % (95% CI) | | 4.0 (2.9-5.3) | 9.2 (7.0-11.7) | 13.9 (10.6-17.5) | 20.3 (15.7-25.4) | 22.7 (17.2-28.7) | 1.50 (0.95-2.35) P=0.081 | 1.39 (0.88-2.19) P=0.155 |
| CMRF(+, number >=2) /SLD(-) | No. at risk | 1,223 | 909 | 151 | 102 | 52 | 27 | | |
| | Cumulative % (95% CI) | | 2.4 (1.6-3.4) | 10.9 (8.1-14.1) | 17.1 (12.7-21.9) | 24.5 (18.5-31.0) | 25.6 (19.3-32.4) | 1.59 (1.01-2.50) P=0.048 | 1.38 (0.87-2.20) P=0.172 |
| CMRF(+, number 1) /SLD(+) | No. at risk | 603 | 467 | 86 | 69 | 38 | 23 | | |
| | Cumulative % (95% CI) | | 2.5 (1.4-4.0) | 4.5 (2.7-6.9) | 7.1 (3.7-11.8) | 16.2 (9.2-25.0) | 18.1 (10.5-27.4) | 1.03 (0.60-1.77) P=0.913 | 1.16 (0.68-2.00) P=0.586 |
| CMRF(+, number >=2) /SLD(+) | No. at risk | 1,578 | 1,222 | 150 | 104 | 74 | 56 | | |
| | Cumulative % (95% CI) | | 2.5 (1.8-3.4) | 9.3 (6.7-12.4) | 12.9 (9.1-17.5) | 19.5 (14.0-25.6) | 21.4 (15.5-27.9) | 1.27 (0.80-2.00) P=0.314 | 1.34 (0.85-2.13) P=0.213 |

## Slide 2
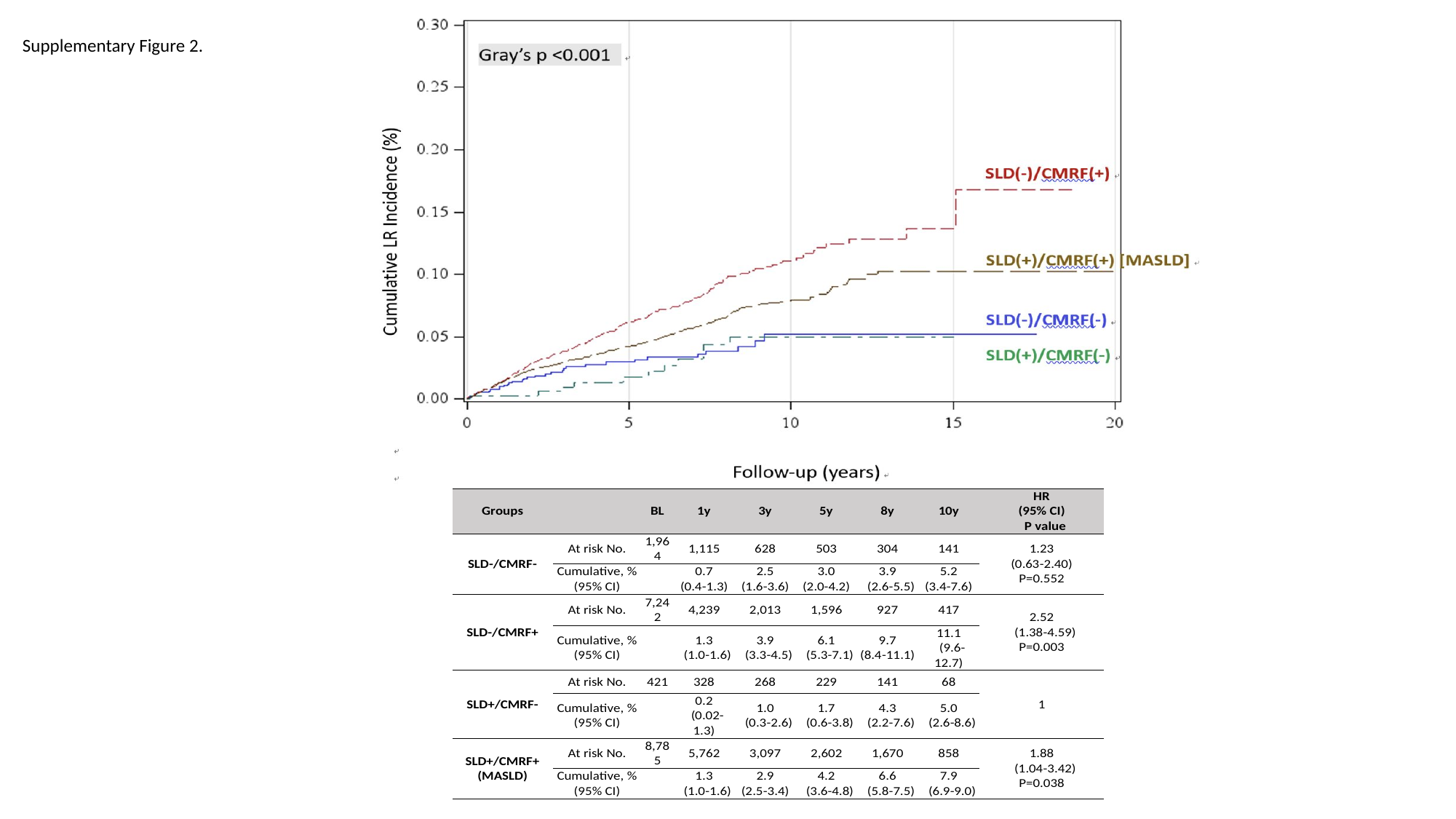

Supplementary Figure 2.
