# Supplementary material for: Impact of Cardiometabolic Risk Factors and Steatotic Liver Disease on Liver‐Related Outcomes in Patients With Chronic Hepatitis C After Curative Antiviral Therapy
Source: Kaohsiung J Med Sci. 2026 Apr 22:e70214. Online ahead of print. doi: 10.1002/kjm2.70214 (PMC13399790; doi:10.1002/kjm2.70214)
Supplement: Supplementary file 2 — Table S1: Disease code (ICD‐9‐CM and ICD‐10) for major outcomes and competing risk. Table S2: Baseline characteristics and incidence of liver related outcomes of SLD and non‐SLD patients before and after propensity score matching (PSM). [file KJM2-9999-e70214-s001.docx]

Supplementary Table 1. Disease code (ICD-9-CM and ICD-10) for major outcomes and competing risk

| **Major outcomes** | ICD 9-CM / ICD-10 | NHIRD Source |
| --- | --- | --- |
| Hepatocellular Carcinoma (HCC) | 155.0  /C22.0, C22.8 | Cancer registry |
| Decompensated Liver Cirrhosis  (DLC) | 571.2, 571.5, 571.6  /K70.2-K70.31, K74.1-K74.69 | Catastrophic illness |
| Liver-Related Outcomes (LRO) | Including HCC and DLC |  |
| **Competing risk** |  |  |
| Death |  | *Death registry* |
| Liver transplant  (LT) | V42.7, 75020A, 75020B, Z94.4 | Catastrophic illness |

Supplementary Table 2. Baseline characteristics and incidence of liver related outcomes of SLD and non-SLD patients before and after Propensity Score Matching (PSM)

|  | **Before PSM** | | | | **After PSM** | | | |
| --- | --- | --- | --- | --- | --- | --- | --- | --- |
|  | **Total** | **SLD-** | **SLD +** | **P value** | **Total** | **SLD-** | **SLD +** | **P value** |
| No. of patients | 21,972 | 11,383 | 10,589 |  | 18,412 | 9,206 | 9,206 |  |
| Age (years) | 60.0±12.6 | 62.0±12.6 | 57.8±12.2 | <0.001 | 59.3±11.8 | 59.3±11.9 | 59.3±11.8 | 0.883 |
| ≤65 | 14,338 (65.3) | 6,689 (58.8) | 7,649 (72.2) | <0.001 | 12,648 (68.7) | 6,357 (69.1) | 6,291 (68.3) | 0.294 |
| >65 | 7,634 (34.7) | 4,694 (41.2) | 2,940 (25.2) |  | 5,764 (31.3) | 2,849 (31) | 2,915 (31.7) |  |
| Sex |  |  |  |  |  |  |  |  |
| Male | 9,472 (43.1) | 4,777 (42.0) | 4,695 (44.3) | <0.001 | 7,887 (42.8) | 3,907 (42.4) | 3,980 (43.2) | 0.277 |
| Female | 12,500 (56.9) | 6,606 (58.0) | 5,894 (55.7) |  | 10,525 (57.2) | 5,299 (57.6) | 5,226 (56.8) |  |
| ***Liver-related factors*** | | | | | | | | |
| Albumin (mg/dL) | 4.2±0.4 | 4.2±0.4 | 4.2±0.4 | <0.001 | 4.2±0.4 | 4.2±0.4 | 4.3±0.4 | 0.825 |
| >3.5 | 15,864 (72.2) | 8,636 (75.9) | 7,228 (68.3) | <0.001 | 13,271 (72.1) | 6,941 (75.4) | 6,330 (68.8) | <0.001 |
| 2.8-3.5 | 854 (3.9) | 564 (5.0) | 290 (2.7) |  | 616 (3.4) | 339 (3.7) | 277 (3) |  |
| <2.8 | 5,254 (23.9) | 2,183 (19.2) | 3,071 (29.0) |  | 4,525 (24.6) | 1,926 (20.9) | 2,599 (28.2) |  |
| AFP(ng/mL) | 11.9±132.0 | 13.3±176.3 | 10.5±49.7 | 0.122 | 10.7±64.9 | 10.6±76.4 | 10.7±50.6 | 0.912 |
| AST (U/L) | 70.0±57.2 | 66.1±58.0 | 74.2±56.1 | <0.001 | 68.9±55.3 | 62.0±52.6 | 75.7±57.0 | <0.001 |
| ≤80 | 15,734 (71.6) | 8,495 (74.6) | 7,239 (68.4) | <0.001 | 13,307 (72.3) | 7,113 (77.3) | 6,194 (67.3) | <0.001 |
| >80 | 6,238 (28.4) | 2,888 (25.4) | 3,350 (31.6) |  | 5,105 (27.7) | 2,093 (22.7) | 3,012 (32.7) |  |
| ALT (U/L) | 95.4±93.4 | 75.7±77.6 | 116.7±103.7 | <0.001 | 95.5±92.1 | 74.1±76.2 | 116.8±101.3 | <0.001 |
| ≤80 | 12,823 (58.4) | 7,943 (69.8) | 4,880 (46.1) | <0.001 | 10,735 (58.3) | 6,545 (71.1) | 4,190 (45.5) | <0.001 |
| >80 | 9,149 (41.6) | 3,440 (30.2) | 5,709 53.9) |  | 7,677 (41.7) | 2,661 (28.9) | 5,016 (54.5) |  |
| Platelet (x10^3^/μl) | 177.7±63.5 | 174.0±64.7 | 181.7±62.0 | <0.001 | 180.4±62.6 | 182.4±63.7 | 178.3±61.4 | <0.001 |
| Creatinine (mg/dL) | 1.10±1.45 | 1.18±1.63 | 1.02±1.23 | <0.001 | 1.10±1.46 | 1.16±1.63 | 1.03±1.26 | <0.001 |
| eGFR (ml/min/1.73m^2^) | 88.0±29.9 | 86.4±31.4 | 89.6±28.2 | <0.001 | 88.6±29.9 | 88.7±31.4 | 88.6±28.5 | 0.856 |
| ≥60 | 19,342 (88.0) | 9,787 (86.0) | 9,555 (90.2) | <0.001 | 16,345 (88.8) | 8,118 (88.2) | 8,227 (89.4) | 0.011 |
| <60 | 2,630 (12.0) | 1,596 (14.0) | 1,034 (9.8) |  | 2,067 (11.2) | 1,088 (11.8) | 979 (10.6) |  |
| FIB-4 | 3.08±2.76 | 3.45±3.08 | 2.69±2.32 | <0.001 | 2.86±2.36 | 2.87±2.31 | 2.85±2.42 | 0.625 |
| ≤3.25 | 14,960 (68.1) | 7,035 (61.8) | 7,925 (74.8) | <0.001 | 13,115 (71.2) | 6,502 (70.6) | 6,613 (71.8) | 0.071 |
| >3.25 | 7,012 (31.9) | 4,348 (38.2) | 2,664 (25.2) |  | 5,297 (28.8) | 2,704 (29.4) | 2,593 (28.2) |  |
| Liver cirrhosis |  |  |  |  |  |  |  |  |
| No | 16,977 (77.3) | 8,616 (75.7) | 8,361 (79.0) | <0.001 | 14,462 (78.5) | 7,234 (78.6) | 7,228 (78.5) | 0.914 |
| Yes | 4,995 (22.7) | 2,767 (24.3) | 2,228 (21.0) |  | 3,950 (21.5) | 1,972 (21.4) | 1,978 (21.5) |  |
| Antivirals |  |  |  |  |  |  |  |  |
| DAA | 14,887 (67.8) | 8,323 (73.1) | 6,564 (62.0) | <0.001 | 12,282 (66.7) | 6,480 (70.4) | 5,802 (63.0) | <0.001 |
| Peg-IFN | 7,085 (32.2) | 3,060 (26.9) | 4,025 (38.0) |  | 6,130 (33.3) | 2,726 (29.6) | 3,404 (37.0) |  |
| ***Outcomes*** | | | | | | | | |
| **Liver-related outcomes**  Person-years | 71,000 | 30,983 | 40,017 |  | 60,562 | 26,628 | 33,934 |  |
| Follow-up (years) |  |  |  |  |  |  |  |  |
| Mean ± SD | 3.2±3.8 | 2.7±3.4 | 3.8±4.1 | <0.001 | 3.3±3.8 | 2.9±3.5 | 3.7±4.0 | <0.001 |
| No. (%) | 745 (3.4) | 422 (3.7) | 323 (3.1) | <0.001 | 611 (3.3) | 305 (3.3) | 306 (3.3) | 0.960 |
| Annual incidence  (per 10,000 PYs) | 104.9 | 136.2 | 80.7 | <0.001 | 100.9 | 114.5 | 90.2 | 0.003 |
| **Decompensated LC**  Person-years | 72,811 | 31,965 | 40,846 |  | 62,105 | 27,389 | 34,716 |  |
| Follow-up (years) |  |  |  |  |  |  |  |  |
| Mean ± SD | 3.3±3.8 | 2.8±3.5 | 3.9±4.1 | <0.001 | 3.4±3.9 | 3.0±3.6 | 3.8±4.1 | <0.001 |
| No. (%) | 53 (0.2) | 35 (0.3) | 18 (0.2) | <0.001 | 42 (0.2) | 27 (0.3) | 15 (0.2) | 0.167 |
| Annual incidence  (per 10,000 PYs) | 7.3 | 10.9 | 4.4 | 0.001 | 6.8 | 9.9 | 4.3 | 0.008 |
| **HCC**  Person-years | 71,104 | 31,048 | 40,055 |  | 60,638 | 26,674 | 33,964 |  |
| Follow-up (years) |  |  |  |  |  |  |  |  |
| Mean ± SD | 3.2±3.8 | 2.7±3.4 | 3.8±4.1 | <0.001 | 3.3±3.8 | 2.9±3.5 | 3.7±4.0 | <0.001 |
| No. (%) | 702 (3.2) | 395 (3.5) | 307 (2.9) | <0.001 | 575 (3.1) | 283 (3.1) | 292 (3.2) | 0.901 |
| Annual incidence  (per 10,000 PYs) | 98.7 | 124.0 | 76.6 | <0.001 | 94.8 | 106.1 | 86.0 | 0.012 |

Note: Note: SLD: steatotic liver disease; MASLD: metabolic dysfunction-associated steatotic liver disease; CMRFs: cardiometabolic risk factors; HCC: hepatocellular carcinoma; FIB-4: fibrosis-4 index; eGFR: estimated glomerular filtration rate; AST: aspartate aminotransferase; ALT: alanine aminotransferase; AFP: alpha fetoprotein; HDL-C: high density lipoprotein cholesterol; LDL-C: low density lipoprotein cholesterol; HbA1c: glycated hemoglobin; DAA: directly acting antiviral agent; IFN: interferon-based therapy; PYs: person-years.
